# Workplace violence, work characteristics, and seniority levels among nurses: a cross-sectional study

**DOI:** 10.1186/s12912-025-02887-3

**Published:** 2025-03-13

**Authors:** Hui-Ling Yang, Ju-Chun Tai, Chia-Hui Wang, Yuh-Kae Shyu, Kuei-Ru Chou, Li-Chung Pien

**Affiliations:** 1 Department of Nursing, College of Medicine, Fu Jen Catholic University, New Taipei City, Taiwan; 2 Tri-Service General Hospital OSCE Center & CoS TEAM, Taipei, Taiwan; 3 School of Nursing, College of Nursing, Taipei Medical University, Taipei, Taiwan; 4 Department of Nursing, Taipei Medical University-Shuang Ho Hospital, New Taipei City, Taiwan; 5 Research Center in Nursing Clinical Practice, Wan Fang Hospital Taipei Medical University, Taipei, Taiwan; 6 Psychiatric Research Center, Taipei Medical University Hospital, Taipei, Taiwan; 7 Research Center for Neuroscience, Taipei Medical University, Taipei, Taiwan; 8 Department of Nursing, College of Medicine and Hospital, National Cheng Kung University, Tainan, Taiwan; 9 Psychiatric Research Center, Wan Fang Hospital, Taipei Medical University, Taipei, Taiwan

**Keywords:** Workplace violence, Nursing staff, Psychological job demand, Workplace justice

## Abstract

**Background:**

Workplace violence (WPV) is a widespread phenomenon in healthcare systems and an increasingly severe occupational health and safety issue. This study aimed to analyze the prevalence of WPV among novice and senior nurses and to explore the associations between this factor and various types of workplace violence, workplace characteristics, and professional seniority levels while simultaneously identifying relevant risk factors.

**Methods:**

The participants in this cross-sectional descriptive survey study were recruited via a convenience sampling approach between February and May 2021. The sample ultimately included 1000 full-time registered nurses who were recruited from a medical center in Taiwan. A self-administered questionnaire was used to collect participants’ experiences of workplace violence. A variety of data were collected, including demographic characteristics, work characteristics, WPV experiences, job control, workplace justice, and psychological job demands. Descriptive statistics and multivariable logistic regression were used in this research.

**Results:**

Nearly 50% of the nurses experienced at least one episode of workplace violence in the last 12 months. Among the types of workplace violence, verbal violence had the highest prevalence, followed by physical violence. High psychological job demands and low workplace justice were associated with workplace violence. After adjustment for demographic characteristics and psychosocial work conditions, novice nurses were more likely to experience workplace violence, particularly psychological violence, than senior nurses were.

**Conclusions:**

Workplace violence is prevalent among Taiwanese nurses, in which context novice nurses are particularly vulnerable. Addressing high levels of psychological job demands and enhancing workplace justice can help mitigate workplace violence. Effective policies are needed to empower nurses and reduce violence.

**Impact:**

Our findings highlight the persistence of workplace violence among nurses, indicating a need to promote healthier work environments. These results offer insights that nurse leaders and policymakers can use to improve workplace characteristics by promoting work autonomy, establishing a safe culture, and ensuring effective violence management, thereby potentially mitigating nurses’ exposure to workplace violence and reducing their turnover intentions.

## Introduction

Workplace violence (WPV) is a widespread phenomenon in healthcare systems and has become an increasingly serious occupational health and safety problem in workplaces. Occupational Health Safety Network (OHSN) WPV injury surveillance data indicated a continuous increase in the annual prevalence of WPV and a 23% annual increase in the mean risk [1]. According to the World Health Organization (WHO), the International Labor Office [2], the International Council of Nurses (ICN), and the Public Services International (PSI), a commonly used definition of WPV focused on “incidents where a staff member is abused, threatened or assaulted in circumstances related to their work, including commuting to and from work, involving an explicit or implicit challenge to their safety, well-being, or health” [3]. WPV is generally divided into the following four major categories: verbal violence (e.g., abusive language, verbal harassment, and cynical comments), psychological violence (e.g., threats, intimidation, discrimination, exclusion, and harassment), physical violence (e.g., beating, kicking, pushing, and pulling), and sexual harassment (e.g., sexually suggestive and inappropriate behaviors) [4].

Nurses account for the greatest proportion of healthcare professionals; however, the prevalence of WPV is 1.7 times greater among nurses than among other healthcare workers [1], and an estimated 17.2% of nurses leave their positions annually as a result of the impact of such incidents [5]. Nurses are caregivers who come into direct contact with patients and are extensively involved in care provision, and patients may participate in WPV behaviors because of disease-related impairment of their mental state or long-term physical and mental discomfort [2, 6]. Furthermore, the prevalence of WPV behavior may increase due to the dissatisfaction of patients’ relatives with the healthcare environment or the services provided [2]. Retrospective studies that have analyzed the prevalence of WPV experienced by nurses in different countries have revealed that the prevalence of WPV reported by nurses ranges from 12 to 85.0% [7]. Previous studies have reported that the risk factors for WPV among nursing staff include shift work [8, 9], long working hours/heavy workloads [10], and low levels of workplace justice [11]. Furthermore, poor communication skills and poor WPV management training increase the risk of WPV [10].

WPV can have adverse psychological and emotional effects on nursing staff [5], such as gastrointestinal symptoms, headaches, anxiety, sleep disorders, depression, and fatigue [2, 6, 12]. In hospitals, WPV reduces nursing staff’s work motivation, job satisfaction, and quality of care [2], thereby leading to job burnout [6] and increasing the workforce turnover rate [2, 13], ultimately affecting the stability of the healthcare environment. In light of this, in addition to the higher prevalence of WPV among nursing staff, the impact of WPV on the healthcare system and patient safety should not be overlooked.

Workplace violence (WPV) has profound and severe adverse effects on healthcare providers, patients, and the overall healthcare system. While most studies on this subject have examined the prevalence, type, and source of WPV among nurses [2, 10], Baharum et al. (2023) highlighted the critical influence of nursing experience—specifically in terms of differences between novice and senior nurses—on professional practice and individuals’ vulnerability to WPV [14]. Seniority in nursing is often defined on the basis of nurses’ years of experience and the corresponding implications for practice. In Taiwan, for example, nurses who have two years of experience or fewer following the postgraduate year (PGY) nursing training program, which is supported by the Ministry of Health and Welfare (MOHW), are categorized as novice nurses, while those who have more than two years of such experience are classified as senior nurses [14, 15]. The unique risk factors and characteristics associated with WPV among novice nurses differ significantly from those pertaining to their senior counterparts. However, few previous studies have explored these distinctions. Novice nurses often require enhanced support resources as well as improved communication and coping skills to navigate these challenges effectively [16]. It is thus essential to explore the specific vulnerabilities exhibited by this population, as reducing WPV prevalence among novice nurses can not only increase their job satisfaction but also help decrease turnover rates, ultimately contributing to the stability of nursing teams [16].

Workplace violence (WPV) poses a significant challenge in healthcare settings; namely, factors such as job control, psychological job demands, and workplace justice play critical roles in the prevalence and impact of this phenomenon. According to Karasek’s job demand-control model, environments that feature high levels of job demand and low levels of job control are classified as high-strain jobs, which entail significant risks of psychological and physical distress. Research has indicated that roles that are characterized by high levels of job demands, such as heavy workloads and understaffing, are key contributors to WPV and are more likely to lead to violent incidents than are roles that feature lower levels of job demands [17]. Conversely, healthcare workers (HCWs) who have greater job control are less likely to experience WPV [18, 19]. Additionally, workplace justice can improve job satisfaction and psychological well-being and reduce the likelihood of conflict and aggression. Conversely, a lack of workplace justice damages morale and elicits resentment, thus increasing the risk of turnover and WPV [18, 20]. Therefore, exploring these variables in the context of workplace factors is crucial with regard to efforts to understand and address WPV.

What are the differences in the prevalence and types of workplace violence (WPV) experienced by novice and senior nurses, and what workplace characteristics and risk factors contribute to these disparities? Addressing these questions is crucial, as WPV poses significant challenges for nursing staff with different levels of seniority. Reducing WPV incidence, particularly among novice nurses, enhances job satisfaction, lowers turnover rates, and fosters stable and effective nursing teams. Understanding these distinctions provides a foundation for developing targeted interventions to mitigate WPV, improve nurse retention, and promote a resilient nursing workforce.

## Aim of the study

The primary aim of this study was to investigate the prevalence of workplace violence (WPV) among novice and senior nurses, particularly by highlighting differences in their vulnerability to various types of WPV. Furthermore, this study aimed to analyze the associations among different types of WPV, workplace characteristics, and the professional seniority levels exhibited by nurses, who were divided into novice nurses (i.e., those with ≤ 2 years of experience) and senior nurses (i.e., those with > 2 years of experience). By identifying the risk factors associated with WPV, the research seeks to provide valuable insights into the unique challenges that nurses face at different stages of their careers. The authors hypothesized that (1) novice nurses experience more WPV incidents than senior nurses and (2) novice nurses have a greater risk of experiencing all types of WPV than senior nurses.

## Methods

### Study design and sampling

This study followed the Strengthening the Reporting of Observational Studies in Epidemiology (STROBE) guidelines for cross-sectional studies. In this cross-sectional survey study, a convenience sampling approach was employed to recruit 1,000 nurses (including 933 women and 67 men) from a medical center in New Taipei City, Taiwan, between February and May 2021. As the hospital on which this study focuses features a total nursing population of 1,000 nurses, we conducted a census survey of all nurses. The adequacy of the sample size was evaluated. This sample size was determined to be adequate in light of the guidelines for logistic regression sample size calculation proposed by Bujang [21], who recommended *n* = 100 + 50(i), where i is the number of independent variables. Since eight independent variables (i.e., sex, level of education, work shift, work hours, job control, psychological job demands, workplace justice, and seniority) were included in our study, the minimum required sample size was 500. Therefore, our sample of 1,000 nurses was more than sufficient for the planned analyses. The hospital has 977 beds, including general and specialized care units, covering various specialties and patient care settings. The inclusion criteria used in this research focused on full-time nurses aged > 20 years who provided direct patient care. The exclusion criteria pertained to (1) nurses who were on extended leave (e.g., maternity leave or sick leave); (2) nurses who worked part-time or were temporary nurses; (3) nurses who were unable to complete the questionnaire independently; and (4) nurses who did not provide direct patient care (e.g., head nurses). Additionally, incomplete questionnaires that featured missing responses were excluded from the analyses. The participants were categorized into senior nurses and novice nurses according to seniority. After the postgraduate year (PGY), nursing training programs in Taiwan—supported by the Ministry of Health and Welfare (MOHW)—last for two years [15]; thus, participants with ≤ 2 years of experience were classified as novice nurses, and those with > 2 years of experience were classified as senior nurses. The data were collected using paper-based questionnaires, which were distributed and completed at the hospital. The researchers personally visited the hospital to explain the study and recruit participants.

### Measurements and variables

The demographic data, including marital status, age, level of education, and seniority, were self-reported. WPV experience was evaluated using one-item questions for each type of WPV, asking participants whether they had ever experienced any of the four types of WPV (physical violence, verbal violence, psychological violence, or sexual harassment) in the past 12 months; the response options were “yes” or “no.” Participants could select multiple types of WPV if they had experienced more than one type simultaneously. We classified participants who had experienced at least one type of WPV, regardless of the specific type(s), as having experienced “any WPV.” Previous studies have used an identical classification system for WPV types [12, 22].

Information on work characteristics, including rotation work shifts, the number of work hours, job control, psychological job demands, and workplace justice, was collected via a self-report questionnaire. The participants were asked whether their work involved fixed/day shifts or night/rotating shifts. Data concerning the average number of hours they had worked per week over the past month were also collected; on this basis, the nurses were divided into two categories (i.e., those who worked ≤ 48 h/week and those who worked > 48 h/week). The 48-hour threshold was selected on the basis of Taiwan’s Labor Standards Act (Article 36), which mandates at least one rest day every seven days [23]. For workers who work six days per week, the total number of standard maximum working hours is 48 (6 days × 8 h per day). This threshold is also in line with categorizations that have been used in previous studies [22, 24].

Job control and psychological job demands were measured via the Chinese version of the Job Content Questionnaire (C-JCQ), which is based on Karasek’s job strain model [25, 26]. Job control indicates the amount of autonomy and discretion that an employee has over their work tasks and processes. Psychological job demands are related to the mental requirements of the job, such as workload, intellectual demands, and time pressure [22, 26]. Job control is measured using nine items, including six for skill discretion and three for decision-making authority. Psychological job demands are measured on a nine-item scale. Both the job control and psychological job demand scales exhibited high reliability; namely, the corresponding Cronbach’s α coefficients were 0.80 and 0.82, respectively [27]. Analyzing job control and psychological job demands as independent constructs allows researchers to obtain a nuanced understanding of their individual and combined effects on WPV and job stress and is consistent with previous research methodologies [22, 27].

Workplace justice was assessed via a nine-item scale that measured employees’ perceptions of fairness in their work environment [22, 28–30]. This scale evaluated four aspects of workplace justice: distributive justice (i.e., fairness in terms of the allocation of rewards and resources), procedural justice (i.e., fairness in terms of organizational processes and decisions), informational justice (i.e., the adequacy and truthfulness of information), and interpersonal justice (i.e., respect and dignity in treatment). Specifically, the nine items included in the scale assessed trust between supervisors and employees, the reliability of the information provided by supervisors, the transparency of communication, employees’ influence on management decisions, fairness in terms of work arrangements, fairness in terms of reward management, fairness in terms of performance evaluation, information provision during decision-making processes, and the respect provided by supervisors. Each item was scored on a 4-point Likert scale ranging from 1 (strongly disagree) to 4 (strongly agree). Items that were listed in the opposite format were reverse-scored, and the total score indicated the level of perceived workplace justice. The scale exhibited a high level of internal reliability, as indicated by a Cronbach’s α value of 0.87 [29]. The total scores were then ranked and divided into tertiles (i.e., low, medium, and high) on the basis of their distribution in our sample, which was in line with the approaches employed in previous research [22, 28, 30].

### Data analysis

The demographic characteristics, work characteristics, and WPV experiences of all participants were analyzed. The demographic characteristics, prevalence of the four types of WPV, and work characteristics stratified by seniority were determined. Furthermore, multivariable logistic regression was used to analyze the associations between various demographic and work characteristics and the types of WPV. Age was not included in the multivariable logistic regression model to avoid potential bias, as age is highly correlated with clinical experience and seniority, potentially resulting in multicollinearity issues. Given that age and psychosocial work conditions (job control, psychological job demands, and workplace justice) have been revealed by previous studies to be correlated with WPV [31, 32], these variables were controlled for and included as confounding factors in the regression models. Since less than 50 participants reported experiencing sexual harassment, this parameter was excluded from the multivariable logistic regression model. All analyses were performed with SPSS 23.0 (IBM, Armonk, NY, USA).

### Ethical considerations

The study was reviewed and approved by the Institutional Review Board of Taipei Medical University (IRB number: N202011044). The questionnaires were distributed by independent researchers to the nursing staff and collected after they were completed anonymously. The independent researchers briefly explained the study’s aims to the participants, and those who agreed to participate provided signed informed consent forms.

## Results

A total of 936 female and 67 male nurses completed the questionnaires. After examination of the responses, three female participants were excluded due to missing responses. Overall, a total of 1000 participants were included in the statistical analysis.

Regarding the demographic characteristics of the participants in this study, the majority of the participants were female (93.3%) and single, widowed, or divorced (74.4%); furthermore, 68.6% of the participants held a university degree or above. Novice nurses (those with ≤ 2 years of seniority) were predominantly younger (100% aged 20–30 years) and more likely to work night shifts or rotating shifts (76.6%) than senior nurses (those with > 2 years of seniority, 84.3%). Additionally, 45.5% of novice nurses worked more than 48 h per week, in comparison with 34.6% of senior nurses (Table 1).

**Table 1:** Demographic and work characteristics of the study subjects (*N* = 1000)

	seniority ≤ 2 years (*n* = 154)		seniority > 2 years (*n* = 846)		Total (*N* = 1000)	
	*n*/mean	%/SD	*n*/mean	%/SD	*n*/mean	%/SD
Demographic characteristics						
Sex						
Female	142	92.2%	791	93.5%	933	93.3%
Male	12	7.8%	55	6.5%	67	6.7%
Marital status						
Single/Widowed/Divorced	151	98.1%	593	70.1%	744	74.4%
Married/Cohabitating	3	1.9%	253	29.9%	256	25.6%
Age (years)						
20–30	154	100.0%	466	55.1%	620	62.0%
31–40	0	0	257	30.4%	257	25.7%
41–50	0	0	109	12.9%	109	10.9%
≥ 51	0	0	14	1.7%	14	1.4%
Level of education						
Five-year junior college program	42	27.3%	272	32.2%	314	31.4%
University degree or above	112	72.7%	574	67.8%	686	68.6%
Work shift						
Fixed day shifts	36	23.4%	133	15.7%	169	16.9%
Night/rotating shifts	118	76.6%	713	84.3%	831	83.1%
Work hours						
≤ 48 h/week	84	54.5%	553	65.4%	637	63.7%
> 48 h/week	70	45.5%	293	34.6%	363	36.3%
Workplace violence (any)	54	35.1%	417	49.3%	471	47.1%
Verbal violence	49	31.8%	359	42.5%	408	40.8%
Physical violence	23	14.9%	186	22.0%	209	20.9%
Psychological violence	18	11.7%	189	22.4%	207	20.7%
Sexual harassment	6	3.9%	42	5.0%	48	4.8%
Work characteristics						
Job control	64.99	7.48	66.25	7.44	66.05	7.45
High	51	33.1%	326	38.5%	377	37.7%
Medium	40	26.0%	254	30.0%	294	29.4%
Low	63	40.9%	266	31.4%	329	32.9%
Psychological job demands	20.75	2.87	21.22	3.02	21.15	3.01
Low	65	42.2%	301	35.6%	366	36.6%
Medium	51	33.1%	282	33.3%	333	33.3%
High	38	24.7%	263	31.1%	301	30.1%
Workplace justice	20.92	2.51	20.10	3.28	20.23	3.18
High	28	18.2%	125	14.8%	153	15.3%
Medium	79	51.3%	385	45.5%	464	46.4%
Low	47	30.5%	336	39.7%	383	38.3%

Abbreviations: SD, standard deviation

Note. Regarding levels of education in Taiwan, the “five-year junior college program” is typically offered by junior colleges or vocational schools; “university degree or above” refers to a bachelor’s degree or above

Univariate logistic regression analysis (Table 2) revealed that novice nurses (i.e., those with ≤ 2 years of seniority) were significantly more likely to experience any type of workplace violence (OR = 1.80, 95% CI = 1.26–2.57, *p* <.01) than were senior nurses. The former were also more likely to experience verbal violence (OR = 1.58, 95% CI = 1.10–2.28, *p* <.01), physical violence (OR = 1.61, 95% CI = 1.00–2.58, *p* <.05), and psychological violence (OR = 2.18, 95% CI = 1.30–3.65, *p* <.01). Other workplace characteristics were also significantly associated with workplace violence. Nurses who worked more than 48 h per week were more likely to experience any type of workplace violence (OR = 1.81, 95% CI = 1.40–2.35, *p* <.001), verbal violence (OR = 1.73, 95% CI = 1.33–2.25, *p* <.001), and psychological violence (OR = 1.43, 95% CI = 1.04–1.95, *p* <.05). High levels of psychological job demands were strongly associated with all types of workplace violence, including physical violence (OR = 2.96, 95% CI = 2.03–4.33, *p* <.001) and psychological violence (OR = 2.46, 95% CI = 1.69–3.60, *p* <.001). Nurses who reported low levels of workplace justice were also more likely to experience verbal violence (OR = 2.20, 95% CI = 1.49–3.25, *p* <.001) and psychological violence (OR = 2.37, 95% CI = 1.46–3.86, *p* <.001).

**Table 2:** Univariate logistic regression analysis of factors associated with workplace violence (*N* = 1000)

Demographic/workplace characteristic	Any WPV		Verbal violence		Physical violence		Psychological violence	
	OR	95% CI	OR	95% CI	OR	95% CI	OR	95% CI
Seniority (reference: seniority > 2 years)								
Seniority ≤ 2 years	1.80**	[1.26, 2.57]	1.58**	[1.10, 2.28]	1.61*	[1.00, 2.58]	2.18**	[1.30, 3.65]
Sex (reference: female)								
Male	0.70	[0.42, 1.16]	0.69	[0.41, 1.17]	0.90	[0.48, 1.69]	0.92	[0.49, 1.71]
Marital status (reference: single/widowed/divorced)								
Married/cohabitating	1.03	[0.78, 1.37]	1.16	[0.87, 1.55]	0.76	[0.53, 1.09]	0.94	[0.66, 1.34]
Level of education (reference: five-year junior college program)								
University degree or above	1.37*	[1.05, 1.80]	1.51**	[1.14, 1.99]	1.17	[0.84, 1.64]	1.26	[0.90, 1.77]
Work shift (reference: fixed day shifts)								
Night/rotating shifts	1.21	[0.87, 1.69]	1.23	[0.88, 1.74]	1.64*	[1.04, 2.59]	1.46	[0.94, 2.27]
Work hours (reference: ≤48 h/week)								
> 48 h/week	1.81***	[1.40, 2.35]	1.73***	[1.33, 2.25]	1.40*	[1.02, 1.91]	1.43*	[1.04, 1.95]
Job control (reference: high)								
Medium	0.77	[0.57, 1.05]	0.74	[0.54, 1.02]	0.77	[0.52, 1.14]	0.73	[0.49, 1.07]
Low	1.22	[0.91, 1.64]	1.19	[0.88, 1.60]	1.13	[0.79, 1.60]	0.95	[0.67, 1.36]
Psychological job demands (reference: low)								
Medium	1.83***	[1.34, 2.47]	1.56**	[1.14, 2.13]	1.28	[0.85, 1.92]	1.35	[0.91, 2.02]
High	3.09***	[2.25, 4.24]	2.71***	[1.97, 3.73]	2.96***	[2.03, 4.33]	2.46***	[1.69, 3.60]
Workplace justice (reference: high)								
Medium	1.06	[0.73, 1.53]	1.02	[0.70, 1.51]	0.86	[0.55, 1.35]	0.89	[0.54, 1.48]
Low	2.01***	[1.38, 2.95]	2.20***	[1.49, 3.25]	1.07	[0.68, 1.69]	2.37***	[1.46, 3.86]

Abbreviations: OR, odds ratio; CI, confidence interval; WPV, workplace violence

*p <.05; **p <.01; ***p <.001

After we adjusted for covariates in the multivariable logistic regression model (Table 3), novice nurses remained significantly more likely to experience any type of workplace violence (OR = 1.74, 95% CI = 1.18–2.57, *p* <.01) and psychological violence (OR = 1.99, 95% CI = 1.15–3.43, *p* <.05). However, no significant differences were observed with regard to verbal violence (OR = 1.43, 95% CI = 0.96–2.13) or physical violence (OR = 1.59, 95% CI = 0.97–2.62).

**Table 3:** Multivariable logistic regression analysis of seniority and workplace violence after adjusting for covariates (*N* = 1000)

Demographic/workplace characteristic	Any WPV		Verbal violence		Physical violence		Psychological violence	
	OR	95% CI	OR	95% CI	OR	95% CI	OR	95% CI
Seniority (reference: seniority > 2 years)								
Seniority ≤ 2 years	1.74**	[1.18, 2.57]	1.43	[0.96, 2.13]	1.59	[0.97, 2.62]	1.99*	[1.15, 3.43]

Note: 1. Multivariable logistic regression model adjusted for sex, level of education, work shift, work hours, job control, psychological job demands, and workplace justice

2. Abbreviations: OR, odds ratio; CI, confidence interval; WPV, workplace violence

3. *p <.05; **p <.01; ***p <.001

## Discussion

The present study aimed to analyze the associations among the various types of WPV, workplace characteristics, and different seniority levels as well as the risk factors associated with WPV among nurses. The first main finding was that nearly half of the 1000 nurses had experienced at least one type of WPV in the past 12 months of employment; this observation was generally consistent with the results of previous studies [10, 13, 33]. Approximately 44.6–92.9% of the nurses reported experiencing at least one or more types of WPV [9, 10, 13]. The reasons for the differences in WPV prevalence observed in this context include national culture [7], practicing hospital, the nature of the department (e.g., emergency department or psychiatric ward) [9, 34], work status, rotation work shifts, and the allocation of prevention resources [35].

International organizations and researchers have been increasingly concerned about WPV in recent years. Therefore, the importance of WPV monitoring and prevention should be emphasized. However, studies from various countries have reported that the prevalence of WPV has remained the same (World Health Organization, 2021). The prevalence of WPV from 2017 to 2020 (63%) was reported to be significantly greater than the corresponding prevalence from 2000 to 2016 (51%), thus indicating that the prevalence of WPV is increasing [10].

### WPV types

Regarding the types of WPV, nurses mainly experienced verbal violence rather than physical violence, psychological violence, or sexual harassment, which is consistent with the results of previous studies [10, 13, 33]. According to Kim, Mayer, and Jones (2021), verbal abuse is more common than physical violence. However, hospital notification systems often underestimate the former type of violence [33]. This trend highlights the need for increased awareness and improved reporting mechanisms that can capture the prevalence of verbal violence against nurses accurately. Effective workplace violence (WPV) notification systems are critical with regard to efforts to reduce violence against nurses. A strong reporting culture facilitates accurate data collection, which is essential with respect to the development of targeted interventions and policies that can address WPV. Additionally, when nurses feel supported during the process of reporting incidents and do not fear retaliation, this situation establishes a safer work environment, reduces burnout, and improves patient safety [36]. Therefore, healthcare institutions should prioritize the establishment and maintenance of comprehensive WPV reporting systems and improvements to violence notification systems and procedures with the goals of reducing notification burdens, protecting nurses, and improving the overall quality of care.

This review of previous studies highlights four major factors that may contribute to the high incidence of verbal violence observed in healthcare settings [2, 5]. First, an organizational culture that lacks robust policies and fails to prioritize workplace safety causes verbal violence to be tolerated and underreported. Second, systemic issues within healthcare environments, such as overcrowding, long wait times, and understaffing, exacerbate relevant tensions, thus leading to more verbal violence incidents. Third, insufficient training in conflict resolution and communication skills entails that healthcare workers are unprepared to manage aggressive behavior, thus increasing their vulnerability. Finally, patients and family members often experience heightened levels of stress and anxiety in the context of medical care, which can escalate into verbal aggression. Therefore, addressing these interconnected factors is essential with regard to efforts to establish a safer and more supportive healthcare environment for both staff and patients.

Furthermore, physical violence is usually the most severe form of violence and should be reported to superiors or other administrative departments compared to other forms of violence. However, previous studies have reported that physical violence may be underreported [37]. This is attributable to the fact that the nurses will account for a patient’s condition (cognitive impairment, nervous damage, and psychiatric disease), and they will not hold the patient responsible or feel the need to report the incident, as the effects on the nurses are minor. Some nurses who reported being harassed also worried that the offender would harass them, become violent toward them, threaten them and their family members, or even affect their work [2, 37]. Furthermore, several nurses mistakenly believe that WPV is part of their job and that they have unfortunately been in the wrong place at the wrong time [2]. In clinical practice, verbal warnings or a lack of additional action are common among offenders [9]. According to several studies, nurses may underestimate the frequency of WPV reports [7, 9, 37].

These results indicate that nurses must have appropriate awareness of WPV to decrease its adverse effects. To decrease the prevalence or harm of WPV, nurses’ awareness of, attitudes toward, and ability to cope with WPV should be strengthened [13], and training content and methods should be designed for different types of WPV. This will increase nurses’ motivation and proactive behavior to ensure that all nurses are well protected and work in a safe working environment.

### Workplace characteristics

The nature of nursing work, higher levels of psychological job demands, and lower levels of workplace justice were revealed to be correlated with all types of WPV; these results are similar to those that have been reported in previous studies [38]. The psychological job demands faced by nurses have been reported to be associated with self-rated health (SRH) [30], job control-related stress, job control, job satisfaction, and fatigue [39, 40]. Nursing inherently involves a high-pressure environment that is associated with significant psychological job demands, including heavy workloads, role conflicts, and time constraints. Nurses are required to maintain high levels of motivation and task performance, thereby achieving quality outcomes with minimal errors, which can lead to various challenges to their physical and mental health. Thus, the implementation of policies that can effectively manage psychological job demands is critical with regard to efforts to improve nurses’ overall well-being.

Workplace justice may be a protective factor against turnover, and lower workplace justice may have adverse psychosocial effects on nursing staff. Therefore, establishing a fair system and increasing work autonomy can decrease turnover intentions among nurses [11]. Moreover, WPV reporting systems remain a critical area for improvement. As noted by Huang et al. (2022), many healthcare institutions worldwide lack effective policies to support WPV reporting [7]. Complex and underdeveloped reporting systems have often been identified as barriers to WPV reporting; this limitation hinders the proper documentation and resolution of such incidents. The findings of a cross-sectional study of nurses in five European countries highlighted the importance of improving WPV reporting systems [41]. The study in question revealed substantial underreporting of WPV events; in particular, nurses identified a fear of retaliation, a lack of institutional support, and burdensome reporting procedures as significant barriers to filing reports. That study also indicated that although nurses experienced high rates of WPV, many chose not to report such incidents due to their belief that reporting these events would not lead to meaningful changes or interventions. These results are indicative of global trends in WPV reporting and highlight the need for institutions to simplify reporting procedures and establish more supportive environments that can encourage nurses to come forward without fearing repercussions.

The non-significant association between job control and WPV observed in this study is consistent with the findings reported by Yeh et al. (2020) [19] and Magnavita et al. (2020) [18]. High-stress, dynamic environments such as those associated with nursing and job control alone may be insufficient to mitigate WPV risks due to the unpredictable nature of patient interactions and workplace demands. In tiered care settings that feature strict protocols and limited autonomy, the protective effect of job control may be limited. Yeh et al. (2020) revealed that WPV reduces employees’ sense of job control and increases their turnover intentions by reducing their job satisfaction and perceived control over the work environment [19]. Although job control can reduce stress and reduce turnover, its mitigating effect may be limited in structured healthcare settings that feature minimal autonomy. On the other hand, Magnavitta et al. (2020) emphasized the fact that job control and workplace justice may indirectly influence the impact of WPV by reducing stress and enhancing coping mechanisms [18]. These findings suggest that while job control is essential with respect to efforts to mitigate workplace stress, the limited relevance of this factor in this context highlights the importance of other factors, such as psychological job demands, social support, and workplace justice, in efforts to address WPV. Future researchers should investigate how job control interacts with these factors as well as its role in the complex dynamics underlying WPV in the context of healthcare. Such investigations could inform the development of tailored interventions that can address the multifaceted challenges associated with WPV in caregiving environments.

In summary, an approach that involves addressing both psychological job demands and workplace justice while simultaneously improving WPV reporting systems is essential with regard to efforts to mitigate the impact of WPV on nursing staff. The development of supportive institutional frameworks can enhance not only nurse retention but also the quality of the care delivered to patients.

### Seniority levels of nursing staff

Novice nurses face unique challenges during their transition from education to clinical practice, thus requiring them to engage in substantial learning and adaptation [14, 42]. The process of adjusting to new work environments, hospital systems, and professional roles, alongside the need to manage complex interpersonal dynamics, often imposes significant psychological strain. Limited experience, underdeveloped coping strategies, and workplace stress further exacerbate the difficulty of this transition [43]. These challenges, which are exacerbated by understaffing, frequently result in feelings of inadequacy and fear, thus increasing these nurses’ susceptibility to workplace violence (WPV) [43].

Research has consistently reported that novice nurses experience higher rates of WPV than do their senior counterparts, particularly with respect to psychological violence [8, 10]. Poorer social support, weaker communication skills, and reduced ability to respond to WPV contribute to the heightened vulnerability exhibited by these nurses [42]. Their limited awareness of WPV and lack of confidence in their ability to address incidents often lead them to adopt negative attitudes toward violence management [43]. These factors, alongside the high prevalence of burnout and turnover in this context, highlight the critical need for targeted interventions during this transitional period [16, 42].

To address these issues, nursing managers and healthcare organizations must implement tailored education and training programs to enhance novice nurses’ confidence and skills pertaining to the management of WPV [43]. Hospitals should prioritize the unique needs of novice nurses, particularly by offering training and support systems that address risks related to WPV. The establishment of a supportive environment, complemented by comprehensive prevention and management strategies, can promote positive attitudes toward violence management and improve nurses’ overall safety and well-being. These measures can not only help novice nurses navigate this critical phase but also contribute to the formation of a resilient nursing workforce [16, 42].

Education should focus on equipping novice nurses with strategies that can enable them to manage workplace stress and address violence effectively while reinforcing management principles and procedures pertaining to WPV. The establishment of robust support systems can improve nurses’ attitudes and institutional awareness, as research has highlighted the significant role played by confidence in shaping individuals’ responses to WPV [13, 43]. Moreover, the need to develop the clinical and professional skills necessary to promote independent practice within a team exacerbates psychological strain, thus increasing the difficulty of the adjustment process [14, 42, 43]. Mentorship by senior nurses is another key approach to the task of supporting novice nurses. Professional guidance and mental health support during the transitional phase can mitigate the impact of psychological violence, strengthen coping strategies, and foster positive working relationships [42, 44]. Alongside ongoing WPV education, mentorship represents an essential buffer against stress and promotes resilience. Therefore, future researchers should explore the specific factors that influence WPV among novice nurses and develop more effective prevention and intervention strategies with the goal of nurses’ awareness of and confidence in their ability to manage WPV, which are essential components of efforts to mitigate these risks and establish a supportive environment for their professional growth.

Interestingly, this study did not reveal any significant differences in the rates of verbal and physical violence rates between novice and senior nurses, which may be due to the “healthy worker effect” [4]. Novice nurses who cannot tolerate violence may choose to resign during their probation period. However, the higher vulnerability to psychological violence exhibited by novice nurses, as indicated by the findings of this research, suggests that resignation within the three-month timeframe may be more closely linked to psychological violence rather than to verbal or physical violence. Future researchers should investigate why novice nurses resign within this timeframe and examine whether verbal or physical violence contributes to their decisions in this regard. Correlation surveys could also provide valuable insights into the relationship between WPV exposure and turnover among novice nurses.

### Implications for nursing management

Our findings highlight the persistence of workplace violence among nurses, thus indicating a need to promote healthier work environments. These results offer insights that nurse leaders and policymakers can use to improve workplace characteristics by promoting work autonomy, establishing a safe culture, and ensuring effective violence management, thereby potentially mitigating nurses’ exposure to workplace violence and reducing their turnover intentions.

### Strengths of the study

The main strength of the present study lies in its comprehensive investigation of WPV types and workplace characteristics within health systems, particularly among nursing staff at different seniority levels. While previous research has predominantly focused on WPV prevalence, types, and sources among nursing staff in different units, limited research has investigated the relationship between seniority levels and violent behaviors. Our study revealed that nurses with different seniority levels experience distinct types of WPV, necessitating tailored WPV policies. These findings can help nurse leaders and policymakers enhance workplace conditions by promoting autonomy, fostering a safe culture, and implementing effective violence management, which may mitigate nurses’ exposure to WPV and reduce turnover intention. Another notable strength is the identification of WPV risk factors and the recognition that psychological job demands and workplace justice may be protective factors against WPV. Therefore, developing effective policies to manage psychological job demands and enhance workplace justice can empower nurses and nurture proactive mindsets, contributing to the mitigation of WPV.

### Limitations of the study

Nonetheless, this study has some notable limitations. First, a convenience sampling approach was used, and the participants were recruited exclusively from a medical center in northern Taiwan, which may limit the external validity of the findings of this research. One important limitation of this study is that we did not collect data concerning the sources of workplace violence, thus rendering us unable to differentiate between internal violence from colleagues and external violence from patients/families. Recent research has reported that these two sources of violence have different impacts on nurses’ health outcomes, such that internal workplace violence is more strongly associated with poor health outcomes than is violence from patients and families [45]. Additionally, different types of workplace violence are associated with distinct antecedent factors that require targeted prevention strategies [46]. Furthermore, we did not account for the participants’ specific workplace care settings. Research has reported that in certain settings, such as emergency departments and mental health units, workplace violence (WPV) events are more frequent. This unaddressed variable could have served as a potential confounder, and future researchers should incorporate a clear way of distinguishing among different workplace settings to capture the variability in WPV incidence across healthcare environments more effectively. Moreover, Future surveys may cover more diverse levels of care, including by incorporating region- or hospital-specific differences to capture various nuances of WPV between novice and senior nursing staff more effectively, thus allowing such research to validate the findings of this study. Second, we assessed the occurrence of WPV only on the basis of a binary “Yes/No” question, which limited the depth of the data collected as part of this research. A more nuanced approach, such as utilizing a Likert scale to measure the frequency of WPV events (e.g., weekly, monthly, rarely, or never), would have provided richer and more informative data. This approach would facilitate a more comprehensive understanding of the frequency and severity of WPV incidents among novice and senior nurses.

Additionally, the exclusion of nurses who left the profession during their probationary period—often as a result of their inability to cope with WPV—may have caused the full impact of WPV on nursing staff to be underestimated. The inclusion of these individuals in future studies would offer a more holistic understanding of the consequences of WPV with respect to nurse retention. The COVID-19 pandemic likely altered workplace conditions and patient interactions, thereby potentially influencing the prevalence of WPV during the study period. This contextual factor adds a layer of complexity to the task of interpreting the findings of this research, as the pandemic may have temporarily increased the frequency or reporting of WPV. Although our study offers a timely depiction of WPV during this period, the findings of this research should thus be interpreted cautiously. Another limitation pertains to our division of nurses into different seniority group, i.e., novice nurses (i.e., those with ≤ 2 years of experience) and senior nurses (i.e., those with > 2 years of experience). While this categorization is in line with the postgraduate year (PGY) nursing training program used in Taiwan, we acknowledge that the senior group spans multiple career stages, thereby potentially obscuring trends in nurses’ levels of WPV exposure. Future research featuring larger sample sizes should consider more detailed seniority categories, such as 0–1 years, 1–5 years, and > 5 years, as suggested by Pien [47], to obtain a better understanding of how workplace violence risks evolve across career stages.

Finally, the study featured a cross-sectional design, which limited its ability to establish causal relationships among the predictor variables. The incorporation of nurses’ clinical experience as a variable in future research could provide a more nuanced understanding of the effects of WPV on nurses at various stages of their careers. Future researchers should also differentiate between internal violence from colleagues and external violence from patients/families, as these different sources of workplace violence may require distinct intervention strategies. Understanding the characteristics and impacts of different types of violence could facilitate the development of more targeted and effective prevention measures, particularly since internal workplace violence has been reported to have stronger negative effects on nurses’ health outcomes [45, 46]. In conclusion, addressing these limitations in future studies—by broadening the scope of participant recruitment, refining the measurement of WPV frequency, clarifying the identity of WPV perpetrators, and accounting for clinical experience—would enhance the generalizability and depth of findings pertaining to WPV among healthcare professionals.

## Conclusions

This study revealed the prevalence of WPV and workplace-related factors among Taiwanese nurses. Nearly 50% of the nurses reported experiencing at least one episode of WPV in the last 12 months, highlighting that there is room for improvement in providing a healthy and supportive work environment. In addition, the types of WPV among nursing staff with different seniority levels also differed. For example, the risk of psychological violence for novice nurses is significantly greater than that for senior nurses. Therefore, WPV prevention strategies and education training for nursing staff with different qualifications should be tailored to various needs. Furthermore, psychological work demands and workplace justice can be viewed as protective factors against all types of WPV. If nursing staff can be empowered to reduce their psychological job demands by formulating effective management policies and a fair system can be established to enhance workplace justice, the occurrence of WPV may be reduced. Future research can further verify the correlation and effectiveness of WPV policy interventions regarding workplace characteristics and WPV.

## Data Availability

The data that support the findings of this study are available from the authors, but restrictions apply to the availability of these data, which were used under license for the current study and are not publicly available. However, the data can be obtained from the authors upon reasonable request and with appropriate permissions.

## Abbreviations

WPV: Workplace violence

## References

[1] Groenewold MR, Sarmiento RFR, Vanoli K, Raudabaugh W, Nowlin S, Gomaa A. Workplace violence injury in 106 US hospitals participating in the occupational health safety network (OHSN), 2012-2015. Am J Ind Med. 2018;61(2):157–66. 10.1002/ajim.22798. 29152784 10.1002/ajim.22798

[2] Lim MC, Jeffree MS, Saupin SS, Giloi N, Lukman KA. Workplace violence in healthcare settings: the risk factors, implications and collaborative preventive measures. Annals Med Surg.2022;78:103727. 10.1016/j.amsu.2022.103727 10.1016/j.amsu.2022.103727 PMC9206999 35734684

[3] Richards J. Management of workplace violence victims. Joint Programme on Workplace Violence in the Health Sector, Geneva, 2003.

[4] Harris MA, Kim J, Demers P. Metabolic health measurements of shift workers in a National cross-sectional study: results from the Canadian health measures survey. Am J Ind Med. 2021;64(11):895–904. 10.1002/ajim.23283. 34346078 10.1002/ajim.23283

[5] Al-Qadi MM. Workplace violence in nursing: A concept analysis. J Occup Health. 2021;63(1):e12226. 10.1002/1348-9585.12226. 33960074 10.1002/1348-9585.12226 PMC8103077

[6] Havaei F, Astivia OLO, MacPhee M. The impact of workplace violence on medical-surgical nurses’ health outcome: A moderated mediation model of work environment conditions and burnout using secondary data. Int J Nurs Stud. 2020;109:103666. 10.1016/j.ijnurstu.2020.103666. 32592889 10.1016/j.ijnurstu.2020.103666

[7] Huang L, Chang H, Peng X, Zhang F, Mo B, Liu Y. Formally reporting incidents of workplace violence among nurses: A scoping review. J Nurs Adm Manag. 2022;30(6):1677–87. 10.1111/jonm.13567. 10.1111/jonm.13567 35213934

[8] Jang SJ, Son YJ, Lee H. Prevalence, associated factors and adverse outcomes of workplace violence towards nurses in psychiatric settings: A systematic review. Int J Ment Health Nurs. 2022;31(3):450–68. 10.1111/inm.12951. 34773361 10.1111/inm.12951

[9] Niu SF, Kuo SF, Tsai HT, Kao CC, Traynor V, Chou KR. Prevalence of workplace violent episodes experienced by nurses in acute psychiatric settings. PLoS ONE. 2019;14(1):e0211183. 10.1371/journal.pone.0211183. 30677077 10.1371/journal.pone.0211183 PMC6345477

[10] Varghese A, Joseph J, Vijay VR, Khakha DC, Dhandapani M, Gigini G, Kaimal R. Prevalence and determinants of workplace violence among nurses in the South-East Asian and Western Pacific regions: a systematic review and meta‐analysis. J Clin Nurs. 2022;31(7–8):798–819. 10.1111/jocn.15987. 34351652 10.1111/jocn.15987

[11] Chin W, Guo YL, Hung YJ, Hsieh YT, Wang LJ, Shiao JS. Workplace justice and intention to leave the nursing profession. Nurs Ethics. 2019;26(1):307–19. 10.1177/0969733016687160. 28095760 10.1177/0969733016687160

[12] Kim HR. Associations between workplace violence, mental health, and physical health among Korean workers: the fifth Korean working conditions survey. Workplace Health Saf 2022.70(3):161–72. 10.1177/21650799211023863 10.1177/21650799211023863 34323126

[13] Cai J, Qin Z, Wang H, Zhao X, Yu W, Wu S, Zhang Y, Wang Y. Trajectories of the current situation and characteristics of workplace violence among nurses: a nine-year follow-up study. BMC Health Serv Res. 2021;21:1–9. 10.1186/s12913-021-07245-y. 34763686 10.1186/s12913-021-07245-y PMC8582131

[14] Baharum H, Ismail A, McKenna L, Mohamed Z, Ibrahim R, Hassan NH. Success factors in adaptation of newly graduated nurses: a scoping review. BMC Nurs. 2023;22(1):125. 10.1186/s12912-023-01300-1jang. 37069647 10.1186/s12912-023-01300-1 PMC10111715

[15] Yin YC. The Two-Year post graduate training program for nurses: implementation status and personal perspectives. J Nurs Res 201360(3):11–6. 10.6224/JN.60.3.11 10.6224/JN.60.3.11 23729336

[16] Matter S, Wolgast KA. Making good use of your limited time: supporting novice nurses. Nurs Clin. 2020;55(1):39–49. 10.1016/j.cnur.2019.10.004. 10.1016/j.cnur.2019.10.004 32005364

[17] Sahiran MN, Minhat HS, Muhamad Saliluddin S. Workplace violence among healthcare workers in the emergency departments in Malaysia. J Health Res. 2022;36(4):663–72. 10.1108/JHR-06-2020-0205.

[18] Magnavita N, Heponiemi T, Chirico F. Workplace violence is associated with impaired work functioning in nurses: an Italian Cross-Sectional study. J Nurs Scholarsh. 2020;52(3):281–91. 10.1111/jnu.12549. 32212311 10.1111/jnu.12549

[19] Yeh TF, Chang YC, Feng WH, Sclerosis M, Yang CC. Effect of workplace violence on turnover intention: the mediating roles of job control, psychological demands, and social support. Inquiry.2020.57:46958020969313. 10.1177/0046958020969313 10.1177/0046958020969313 PMC7750754 33334214

[20] Chen HC, Wang JY, Lee YC, Yang SY. Examining the predictors of turnover behavior in newly employed certified nurse aides: A prospective cohort study. Saf Health Work. 2023;14(2):185–92. 10.1016/j.shaw.2023.04.003. 37389317 10.1016/j.shaw.2023.04.003 PMC10300465

[21] Bujang MA, Sa’at N, Sidik TMITAB, Joo LC. Sample size guidelines for logistic regression from observational studies with large population: emphasis on the accuracy between statistics and parameters based on real life clinical data. Malays J Med Sci 2018 25(4): 122–30. 10.21315/mjms2018.25.4.12 10.21315/mjms2018.25.4.12 PMC6422534 30914854

[22] Lin MW, Wang YT, Cheng Y. Psychosocial work conditions during the COVID-19 pandemic and their influences on mental health risk and intention to leave among public health workers: a cross-sectional and follow-up study in Taiwan. Saf Health Work. 2023;14(4):438–44. 10.1016/j.shaw.2023.10.007. 38187201 10.1016/j.shaw.2023.10.007 PMC10770106

[23] Ministry of Labor. (2024). Labor Standards Act. Laws & Regulations Database of The Republic of China. https://law.moj.gov.tw/ENG/LawClass/LawAll.aspx?pcode=N0030001

[24] Cheng Y, Li YJ, Cheng WJ. Gender-and age-specific associations between psychosocial work conditions and perceived work sustainability in the general working population in Taiwan. PLoS ONE. 2023;18(10):e0293282. 10.1371/journal.pone.0293282. 37878636 10.1371/journal.pone.0293282 PMC10599519

[25] Cheng Y, Luh WM, Guo YL. Reliability and validity of the Chinese version of the job content questionnaire in Taiwanese workers. Int J Behav Med 200310(1): 15–30. 10.1207/s15327558ijbm1001_02 10.1207/s15327558ijbm1001_02 12581945

[26] Karasek R, Brisson C, Kawakami N, Houtman I, Bongers P, Amick B. The job content questionnaire (JCQ): an instrument for internationally comparative assessments of psychosocial job characteristics. J Occup Health Psychol. 1998;3(4):322–55. 10.1037/1076-8998.3.4.322. 9805280 10.1037//1076-8998.3.4.322

[27] Wang YT, Cheng Y. Workplace violence and associations with health outcomes in public health administration agencies in Taiwan: a survey during the COVID-19 pandemic. Taiwan Gong Gong Wei Sheng Za Zhi. 2023;42(1):42–61.

[28] Cheng WJ, Pien LC, Cheng Y. Occupation-level automation probability is associated with psychosocial work conditions and workers’ health: A multilevel study. Am J Ind Med. 2021;64(2):108–17. 10.1002/ajim.23210. 33350480 10.1002/ajim.23210

[29] Cheng Y, Huang HY, Li PR, Hsu JH. Employment insecurity, workplace justice and employees’ burnout in Taiwanese employees: a validation study. Int J Behav Med 201118:391–401. 10.1007/s12529-011-9152-y 10.1007/s12529-011-9152-y 21380932

[30] Pien LC, Cheng WJ, Chou KR, Lin LC. Effect of work–family conflict, psychological job demand, and job control on the health status of nurses. Int J Environ Res Public Health 202118(7):3540. 10.3390/ijerph18073540 10.3390/ijerph18073540 PMC8037057 33805465

[31] Cheng Y, Chen IS, Chen CJ, Burr H, Hasselhorn HM. The influence of age on the distribution of self-rated health, burnout and their associations with psychosocial work conditions. J Psychosom Res 201374(3):213–20. 10.1016/j.jpsychores.2012.12.017 10.1016/j.jpsychores.2012.12.017 23438711

[32] Cheng Y, Chen IS, Burr H, Chen CJ, Chiang TL. Changes in psychosocial work conditions in Taiwanese employees by gender and age from 2001 to 2010. J Occup Health 201355(5):323–32. 10.1539/joh.12-0286-oa 10.1539/joh.12-0286-oa 23812030

[33] Kim S, Mayer C, Jones CB. Relationships between nurses’ experiences of workplace violence, emotional exhaustion and patient safety. J Res Nurs. 2021;26(1–2):35–46. 10.1177/1744987120960200. 35251222 10.1177/1744987120960200 PMC8894792

[34] Lee HL, Han CY, Redley B, Lin CC, Lee MY, Chang W. Workplace violence against emergency nurses in Taiwan: a cross-sectional study. J Emerg Nurs 202046(1):66–e714. 10.1016/j.jen.2019.09.004 10.1016/j.jen.2019.09.004 31733817

[35] Yenealem DG, Woldegebriel MK, Olana AT, Mekonnen TH. Violence at work: determinants & prevalence among health care workers, Northwest Ethiopia: an institutional based cross sectional study. Annals Occup Environ Med 2019.31:1–7. 10.1186/s40557-019-0288-6 10.1186/s40557-019-0288-6 PMC6449966 30992993

[36] Kim S, Lynn MR, Baernholdt M, Kitzmiller R, Jones CB. How does workplace Violence-Reporting culture affect workplace violence, nurse burnout, and patient safety?? J Nurs Care Qual 2023.38(1):11–8. 10.1097/NCQ.0000000000000641 10.1097/NCQ.0000000000000641 36409656

[37] Song C, Wang G, Wu H. Frequency and barriers of reporting workplace violence in nurses: an online survey in China. Int J Nurs Sci. 2021;8(1):65–70. 10.1016/j.ijnss.2020.11.006. 33575447 10.1016/j.ijnss.2020.11.006 PMC7859538

[38] Pien LC, Cheng Y, Cheng WJ. Internal workplace violence from colleagues is more strongly associated with poor health outcomes in nurses than violence from patients and families. J Adv Nurs 201975(4):793–800. 10.1111/jan.13887 10.1111/jan.13887 30375031

[39] Bagheri Hossein Abadi M, Taban E, Khanjani N, Naghavi Konjin Z, Khajehnasiri F, Samaei SE. Relationships between job satisfaction and job demand, job control, social support, and depression in Iranian nurses. J Nurs Res. 2021;29(2):e143. 10.1097/jnr.0000000000000410. 10.1097/jnr.0000000000000410 33156140

[40] Jalilian H, Shouroki FK, Azmoon H, Rostamabadi A, Choobineh A. Relationship between job stress and fatigue based on job demand-control-support model in hospital nurses. Int J Prev Med.2019.10:56. 10.4103/ijpvm.IJPVM_178_17 10.4103/ijpvm.IJPVM_178_17 PMC6528424 31143430

[41] Babiarczyk B, Turbiarz A, Tomagová M, Zeleníková R, Önler E, Sancho Cantus D. Reporting of workplace violence towards nurses in 5 European countries–a cross-sectional study. Int J Occup Med Environ Health. 2020;33(3):325–38. 10.1186/s12912-023-01300-1. 32235948 10.13075/ijomeh.1896.01475

[42] Chao LF, Guo SE, Xiao X, Luo YY, Wang J. A profile of novice and senior nurses’ communication patterns during the transition to practice period: an application of the roter interaction analysis system. Int J Environ Res Public Health 202118(20):10688. 10.3390/ijerph182010688 10.3390/ijerph182010688 PMC8535576 34682434

[43] Chen L, Zhang A, Feng X. Interns’ and junior nurses’ Workplace-Violence perceptions, coping confidence and attitudes towards violence management: A structural equation model analysis. J Clin Nurs. 2024. 10.1016/j.shaw.2023.04.003. Advance online publication. 39520050 10.1111/jocn.17536

[44] Alshawush K, Hallett N, Bradbury-Jones C. The impact of transition programmes on workplace bullying, violence, stress and resilience for students and new graduate nurses: A scoping review. J Clin Nurs. 2022;31(17–18):2398–417. 10.1111/jocn.16124. 34811826 10.1111/jocn.16124

[45] Pien LC, Cheng Y, Cheng WJ, Cheng. Internal workplace violence from colleagues is more strongly associated with poor health outcomes in nurses than violence from patients and families. J Adv Nurs 2019.75(4):793–800. 10.1111/jan.13887 10.1111/jan.13887 30375031

[46] Nowrouzi-Kia B, Isidro R, Chai E, Usuba K, Chen A. Antecedent factors in different types of workplace violence against nurses: A systematic review. Aggress Violent Beh. 2019;44:1–7. 10.1016/j.avb.2018.11.002.

[47] Pien LC, Wang CH, Cheng WJ, Lin YH, Chou KR, Hsu CY. The relationship between resilience and mental health status among nurses with workplace violence experiences: A Cross-Sectional study. Int J Mental Health Nurs 2024.34(1): e13497. 10.1111/inm.13497 10.1111/inm.13497 39710807

